# The influence of adverse childhood experiences and depression on addiction severity among methamphetamine users: exploring the role of perseveration

**DOI:** 10.3389/fpsyt.2024.1382646

**Published:** 2024-05-14

**Authors:** Cheng-Hung Ko, Yung-Chin Lu, Chun-Hung Lee, Yu-Chi Liao

**Affiliations:** ^1^Department of Addiction and Forensic Psychiatry, Jianan Psychiatric Center, Ministry of Health and Welfare (MOHW), Tainan, Taiwan; ^2^Department of Clinical Psychology, Jianan Psychiatric Center, Ministry of Health and Welfare, Tainan, Taiwan; ^3^Department of Addiction Psychiatry, Taoyuan Psychiatric Center, Ministry of Health and Welfare, Taoyuan, Taiwan; ^4^Department of Psychology, College of Medical and Health Science, Asia University, Taichung, Taiwan; ^5^Center for Prevention and Treatment of Internet Addiction, Asia University, Taichung, Taiwan; ^6^Clinical Psychology Center, Asia University Hospital, Taichung, Taiwan

**Keywords:** adverse childhood experiences, methamphetamine use disorder, decision-making, inhibitory control, perseveration

## Abstract

**Aims:**

This investigation aimed to clarify the intricate relationship among depression, cognitive function, adverse childhood experiences (ACEs), and their combined influence on methamphetamine use disorder (MUD).

**Methods:**

Utilizing a battery of psychological tests, this study ascertained the impact of ACEs on the condition of 76 people with MUD who meet the Diagnostic and Statistical Manual of Mental Disorders (DSM-5) criteria, aged 42.17 on average. The Iowa Gambling Task (IGT), Conners’ Continuous Performance-II (CPT-II), the self-report Severity of Dependence Scale (SDS), and the Beck Depression Inventory-II (BDI-II) were used for these evaluations. Individuals involved in the study were categorized into two discrete cohorts, mild (ME) and severe (SE), based on the extent of their ACEs exposure. This study employed the PROCESS regression, the independent t-test andχ2 tests for the analysis.

**Results:**

The findings revealed notable discrepancies in the psychological consequences between the two groups with different degrees of ACEs; however, no substantial differences were observed in the demographic parameters. The SE group exhibited elevated BDI-II scores, more evident indications of MUD, and a higher degree of CPT-II cognitive perseveration. The PROCESS model revealed that cognitive perseveration moderated the impact of depression on ACEs and subjective MUD severity, explaining 20.2% of the variance. The ACEs and depression predicted 28.6% of the variance in MUD symptoms. However, no statistically significant differences were detected between the two groups regarding the parameters in the IGT-2 assessment.

**Conclusions:**

These results indicate that the interaction between cognitive and depressive factors mediates the effect of ACEs on subjective MUD severity but not on MUD symptoms. The ACEs significant impact on mental health severity perception is explained by cognitive and depressive factors. This implies that MUD treatment and rehabilitation should address cognitive dysfunction and developmental trauma.

## Introduction

Drug addiction is a chronic medical condition with complex cognitive, physiological, and behavioral symptoms related to unintentional drug use (1). Illicit drug use remains a major concern in Taiwan. Over the past decade, Taiwan’s criminal justice system has spent over three billion NTD annually on addiction rehabilitation and drug prevention (2). Taiwan has implemented treatment programs to replace punitive approaches to illegal drug use (3, 4). Contrary to the successful control of opioid drug use, the use of methamphetamine in pursuit of heightened pleasure and increased sexual activity (5, 6) poses a significant challenge for individuals in Taiwan attempting to pursue methamphetamine abstinence. Methamphetamine use can lead to legal issues, high-risk sexual behavior, sexually transmitted infections, withdrawal symptoms, psychosis, depression, anxiety, and post-traumatic stress disorder (7, 8). Taiwanese methamphetamine users have a higher all-cause mortality rate than the general population (9). Examining why people use non-opioids may assist in helping the users develop effective addiction-coping mechanisms, which is necessary to address these concerns.

In recent years, there has been growing emphasis on analyzing childhood-related factors to gain insights into the initiation and progression of substance abuse and dependence trajectories. Increasing interest has emerged in the significance of adverse childhood experiences (ACEs) (10). The ACEs encompass a wide range, including abuse (emotional, physical, or sexual), neglect (emotional or physical), severe household dysfunction (e.g., witnessing domestic violence, household member drug use, and parental separation and incarceration), and peer, community, and collective violence (11). These early life adversities, which are prevalent across diverse social and cultural contexts (12), have been associated with detrimental health and sociobehavioral outcomes throughout an individual’s lifespan (13). The likelihood of engaging in illicit drug use and developing addiction increases according to the cumulative number of ACE categories (14, 15). The impact of ACEs on the risk of developing methamphetamine (METH)-associated psychosis was also found to be substantial, with a notable correlation demonstrating an escalation in methamphetamine-associated psychosis corresponding to an increase in the number of ACEs (16). Moreover, increased recurrent methamphetamine use was associated with severe ACEs (17).

As ACEs may predispose individuals to addiction in adulthood (18, 19), it is imperative to comprehend how these early life experiences induce addiction. Adverse Childhood Experiences have been observed to disrupt emotion regulation, resulting in an impaired ability to effectively manage distressing emotions (20) or coping mechanisms with stressful events (21) in a manner that is both healthy and adaptive. Consequently, individuals may resort to substance use as a coping strategy to alleviate and regulate these emotions (22, 23), explains the association between ACEs and Substance Use Disorder (SUD). Recent evidence suggests a symbiotic relationship between methamphetamine use and depression, where each may exacerbate the vulnerability to the other. Compared with other negative emotions, the influence of depression on drug addiction holds greater clinical significance (24). A previous systematic review and meta-analysis also revealed an association between methamphetamine use and comorbid depression (25). Individuals who use methamphetamine are more likely to experience depression compared to those who do not use the substance, indicate that methamphetamine use is a potential risk factor for depression (26). The intricate relationship between substance abuse and depression is a critical area of study within the realm of addiction research. Understanding this relationship is paramount, given the potential for depression to both precede and result from methamphetamine abuse, thereby creating a vicious cycle that complicates treatment and recovery efforts. Moreover, exposure to adversity during critical periods of development is more prone to enduring rather than temporary effects on neurodevelopment (27). Modification of brain neurodevelopment correlates with ACEs, specifically affecting regions such as the prefrontal cortex and the hippocampus (28), which may lead to compromised cognitive functioning (29). Individuals with ACEs exhibit extensive and clinically significant impairments in various cognitive domains, including processing speed, executive function, perceptual reasoning, memory, and verbal comprehension (30). The impact of ACEs on cognitive functioning may extend to later adulthood (31). Cognitive functionality is a potentially valuable area for interventions targeting both clinical and nonclinical individuals who have experienced childhood adversity (32).

Cognitive factors that are explicitly targeted in evidence-based psychotherapies and are malleable, have significant relevance in clinical practice (33). Addiction has been defined as a neurological disorder with remarkable advancements in neuroscience methodologies, and neuropsychological models offer enhanced explanatory frameworks for comprehending addictive behavior (34). These integrated models encompass the Impaired Response Inhibition and Salience Attribution framework (I-RISA) (35) and the Vulnerabilities in Decision-Making model (36). These theoretical frameworks postulate that addiction is associated with poor top-down cognitive control of behavior (executive functions), ultimately impacting the critical stages of the addictive cycle (37). Dysfunctional response inhibition (35); deficits in attentional control, inhibitory control, and behavioral monitoring (38); and impaired decision-making (39) have been generally emphasized as factors that predispose individuals to addiction.

A clear examination of the connections between ACEs and cognition (e.g., inhibitory control and decision-making) in addicted patients (e.g., methamphetamine addicts) has the potential to offer vital insights into strategies for preventing or treating these interrelated risk factors that compromise health. However, despite evidence establishing a connection between ACEs and methamphetamine addiction as well as some possible indications of the underlying mechanisms linking these two factors to adverse neurobiological outcomes, few empirical studies have examined how ACEs uniquely affect neurocognitive function in methamphetamine use disorder (MUD) samples. This study seeks to delve deeper into the interconnected roles of ACEs, depression, and cognitive dysfunction—specifically perseveration, a form of repetitive and rigid thinking—in the severity of methamphetamine addiction. This study categorized individuals with addiction into two distinct groups: Mild exposure (ME) and severe exposure (SE), to identify the factors influenced by childhood adversity. By examining the cumulative impact of these factors, the research aims to uncover the underlying mechanisms that contribute to addiction severity among methamphetamine users. The correlation between ACEs and depression, and their combined effect on addiction severity, necessitates a comprehensive investigation to inform targeted interventions. In addition to investigating executive function and decision-making abilities, this study aimed to incorporate demographic variables and depression levels derived from the literature (16, 24). Given the significant clinical relevance of depression in the context of drug addiction and the potential for ACEs to exacerbate this relationship, this study’s objective is to explore the complex interplay between childhood adversity, depression, perseverative cognitive processes, and addiction severity. By categorizing individuals based on the severity of childhood adversity and examining the influence of depression and cognitive dysfunction, this research aims to establish a predictive model that elucidates the multifaceted dynamics contributing to methamphetamine addiction severity. The findings may offer valuable insights into the development of more effective addiction treatment and prevention strategies, emphasizing the need for addressing mental health disorders and adverse childhood experiences in the context of substance abuse rehabilitation.

## Materials and methods

This cross-sectional study was conducted in two phases. During the initial phase, comprehensive demographic information including age, gender, education, marital status, drug use history, and details of previous suicide attempts was collected. A trained psychologist conducted structured interviews and questionnaires such as the Adverse Childhood Experiences - International Questionnaire (ACE-IQ), the Severity of Dependence Scale (SDS), and the Beck Depression Inventory-II (BDI-II) were collected. During the subsequent phase, a different trained clinical psychologist administered assessments to evaluate executive function and attentional ability using the Conners’ Continuous Performance Test II (CPT-II) and decision-making skills were tested using the Iowa Gambling Task (IGT).

### Participants

To mitigate potential confounding effects of varying substance use, this study focused primarily on individuals with a history of methamphetamine use. Utilizing G*Power (40 for our linear regression analysis, which includes three predictors—childhood adversity, cognitive function, and depression—with a medium effect size (f ^2^ = .16) as suggested by Cohen (41), We calculated that a sample size of 76 is sufficient to achieve a power of 0.8. A total of seventy-six methamphetamine addicts (methamphetamine as the primary drug used in the past and present, polysubstance use: 25%) classified according to the Diagnostic and Statistical Manual of Mental Disorders-Fifth Edition (DSM-5) criteria (the diagnostic instrument commonly employed in clinical or research conditions), as verified by an addiction major psychiatrist, were recruited from the Addiction Treatment Clinics of the Jianan Psychiatric Center between January and December 2022. All participants were outpatients, aged between 22 and 67, did not have severe psychotic disorders or other systemic diseases and completed the first phase of data collection during the initial outpatient consultation. The self-reported average duration of abstinence is approximately 76 days (**Table 1**, range: 7-271 days). The rate of urine-verified abstinence was also 100% at the first visit. Participants who faced difficulties completing the assessment or who could not understand the content of the questionnaires because of intellectual disabilities were excluded from the sample. The researchers informed all participants that their involvement in the study would not affect their legal status. All participants provided written informed consent before the study.

**Table 1:** Independent T-test for SE/ME subgroups.

	Severe Exposure (SE) (ACE >= 4)		Mild Exposure (ME) (ACE < 4)		X^2^/t-test	Effect size
	(n = 45; 59.2%)		(n = 31; 40.8%)			
Variables	M	SD	M	SD	p-value	Cohen’s d/Odds ratio
Gender (F/M)	6/39^a^		2/29 ^a^		.460	–
Age	40.36	10.54	44.81	9.03	.059	–
IQ	89.91	13.06	89.58	17.06	.924	–
Abstinence time	79.20	74.54	70.64	70.48	.677	–
ACE	5.80	1.78	2.06	0.93	<.001	2.63
First use age	28.09	11.48	34.03	14.09	.047	0.46
Sustain use years	13.27	12.80	11.61	9.54	.521	–
Previous suicidal attempt	10/35 ^a^		1/30 ^a^		.023	8.57^b^
IGT-total money	-1182.00	816.38	-1504.83	830.03	.100	–
IGT-T score	43.68	6.64	44.40	6.67	.650	–
Omission	58.60	32.13	66.79	48.37	.378	–
Commission	50.13	10.42	49.86	10.00	.912	–
Reaction time	53.82	11.97	53.75	12.20	.982	–
RTSD	51.37	15.32	47.09	13.54	.214	–
Variability	54.49	14.53	49.45	13.64	.541	–
Detectability	50.10	10.34	50.39	10.70	.907	–
Perseveration	62.25	29.65	50.12	11.55	.016	0.54
Hit RT Block Change	52.02	11.96	49.08	8.82	.421	–
SDS	5.33	3.57	4.10	3.52	.139	–
BDI-II	13.24	11.82	4.55	4.99	<.001	0.96
DSM-5 symptoms	4.98	1.97	3.16	1.29	<.001	1.09

^a^N; ^b^Odds ratio; SE, severe exposure group; ME, mild exposure group; IQ, intelligence score; ACE, adverse childhood experience; IGT-total money, mean total money earned in Iowa Gambling task; IGT-T score, T-score in Iowa Gambling task; Omission, t-score of omission index in CPT-II; Commission, t-score of commission index in CPT-II; Reaction time, t-score of reaction time in CPT-II; RTSD, Hit reaction time standard Error of CPT-II; Variability, t-score of variability index in CPT-II; Detectability, t-score of detectability index in CPT-II; Perseveration, t-score of perseveration index in CPT-II; Hit RT Block Change, t-score of hit reaction time of block change in CPT-II; SDS, severity of dependence scale; BDI-II, Beck depression inventory-II.

### Measures

#### Demographic information

In the demographic questionnaire, age, gender, educational qualifications, employment status, marital status, drug use history, whether the individual met the diagnostic criteria for a psychiatric disorder, age of first-time methamphetamine use, duration of use (defined as the last time taken, minus the first time), and previous suicide attempts were collected.

#### Adverse childhood experiences - international questionnaire

The 29-item ACE-IQ (42) measures exposure to “childhood maltreatment,” “family/household dysfunction,” and “violence outside the home.” Multiple countries are validating this tool, with the trials being part of larger health surveys (11). Participants were asked to answer questions about their first 18 years of age.

Each question has dichotomous (Yes/No) or four-point Likert scale responses from “Never” to “Always” or “Many times.” The ACE-IQ instrument measures exposure to 13 categories of ACEs, which can be combined to determine ACE exposure. To be exposed, at least one ACE category item must be answered yes. Thus, the initial scoring process for each ACE category determines whether participants are “exposed” or “not exposed” to it. Summing the number of ACE categories, the participants considered “exposed” yields an ACE score from 0–13. The Chinese version of the ACEs had a Cronbach’s alpha of 0.83, indicating internal consistency. The “childhood maltreatment,” “family/household dysfunction,” and “violence outside the home” domain subscales had Cronbach’s alpha values of 0.74, 0.62, and 0.60, respectively. The tests and retests showed no differences in response concentration or rank variance. The ACEs had good test-retest reliability (ICC = 0.90), and all three subscales had ICCs between 0.78 and 0.90 (11).

According to previous studies, individuals who have encountered four or more ACEs have a higher likelihood of various health-related outcomes than those who have not (14, 43–45). Participants with ACE scores lower than four were assigned mild exposure (ME), and those with more than or equal to four were assigned severe exposure (SE) (46–48).

#### The Conners’ continuous performance test II

This task is a visual paradigm used for the evaluation of attention and the response inhibition component of executive control. It represents a reliable and objective assessment of diagnostic procedures for attention-deficit/hyperactivity disorder (ADHD) and various other neurological disorders (49).

The CPT-II paradigm has six blocks with three sub-blocks. Targeted and non-targeted stimuli (letters) were randomly presented for 250 ms with varying ISIs within the blocks. A block’s three ISI sub-blocks may be 1, 2, or 4 seconds, and their order varies (50). The CPT-II generates 13 indices, such as correct hits, omission errors, and commission errors for interpretation (50), and this study used T-score formats from computer-generated reports. T-scores above 60 indicated attention issues (49). The current CPT-II split-half reliability ranged from 0.66 to 0.95, indicating good reliability. Test-retest reliability was excellent when individual measures were aggregated into ADHD (0.89) and neurological functioning (0.92) indices (45).

##### Iowa Gambling Task

The Iowa Gambling Task (IGT) involving probabilistic learning via monetary rewards and punishments was created to assess real-world decision-making, where advantageous task performance requires participants to make choices that favor long-term benefits over immediate but potentially more significant rewards to avoid the risk of experiencing substantial losses (51). The present study employed the Iowa Gambling Task™, Version 2 (IGT™2, PAR. Inc.) as an assessment tool to evaluate decision-making abilities mediated by the prefrontal cortex of addicts. The IGT™-2 as the gain-loss structure for clinical is identical to the original IGT (52) but extends the age range by including additional normative data for children and adolescents ages 8 to 17 (53). After receiving clear instructions, participants selected cards from four decks with financial rewards and punishments. This version had 100 selections in five 20-trial blocks (500ms inter-trial interval), displaying demographically corrected T-scores. The retest of IGT blocks showed no difference, with a high Cronbach’s alpha (α=0.83) (54). The construct validity of the IGT may indicate frontal lobe dysfunction (55) or poor decision-making in substance-addicted individuals (52). These findings show modest construct validity of the IGT.

#### The severity of dependence scale

The SDS was developed as a tool specifically designed to assess the degree of dependence experienced by users of different types of substances (56). It includes five items on drug use anxiety and control issues (57). Items 1, 2, and 4 (0 = never, 1 = sometimes, 2 = often, 3 = always, or nearly always), 3 (0 = not at all, 1 = a little, 2 = quite a lot, 3 = a great deal), and 5 (0 = not difficult, 1 = quite difficult, 2 = very difficult, 3 = impossible) were scored on a four-point scale (0 to 3). Higher total SDS scores indicate greater dependence, ranging from 0 to 15. The Chinese version of the SDS has.88 test-retest reliability and Cronbach’s alpha was.75 (58).

Number of DSM-5 Criteria of MUD: The utilization of the DSM-5 criteria for Methamphetamine Use Disorder (MUD) in our study serves as an instrumental measure for assessing the severity of this condition among participants. This assessment is conducted through structured clinical interviews meticulously designed to evaluate the presence and severity of symptoms in alignment with the DSM-5 standards. The DSM-5 criteria, which categorize the severity of the disorder into mild (2-3 criteria), moderate (4-5 criteria), and severe (6 or more criteria). Serving as a fundamental tool for the evaluation of stimulant use disorder, the DSM-5 criteria offer a comprehensive framework to systematically gauge the extent of substance use and its impact.

#### Beck depression inventory-II

The BDI-II (59) has 21 four-point scale items (0 to 3). The BDI-II-C has proven to be reliable by empirical studies. Clinical samples had internal consistency reliability coefficients of.94 (60), while nonclinical samples had reliability coefficients of.88 –.94 (61, 62). Regarding validity, BDI-II-C is significantly correlated with hopelessness, cognitive distortion, suicidal ideation, and health status (60, 61).

### Statistical analysis

The participants’ demographic, neuropsychological, and behavioral scale data are shown as means and standard deviations. Data analysis, including descriptive, independent sample t-tests and χ2 tests, was conducted in SPSS 26.0. Model templates for PROCESS by Hayes in Process 4.1 were used to elucidate the intricate relationships among childhood adversity, cognitive function, and depression status (63). Our analytical model was designed to investigate the direct and indirect effects of ACEs on MUD severity, with particular attention to the roles of cognitive function and depression. Specifically, we posited depression as a mediator that potentially channels the influence of ACEs on MUD severity. Moreover, we explored cognitive function, operationalized through measures of perseveration obtained from the Conners’ Continuous Performance Test II (CPT-II), as both a mediator and a moderator in the relationship between depression and MUD severity. Also, our analysis accounted for potential covariates, including demographic variables (e.g., age, gender, and education), to control for their effects on the relationships of interest. These covariates were selected based on their theoretical relevance and prior research indicating their potential influence on the variables of interest. Continuous variables, such as ACE scores and BDI-II scores, were mean-centered prior to analysis to facilitate the interpretation of interaction effects and to reduce multicollinearity among predictors. The PROCESS macro was employed to estimate the direct and indirect effects within our model, using bootstrap sampling (5,000 samples) to generate 95% confidence intervals for indirect effects. This approach allowed for a robust examination of the hypothesized mediation and moderation effects, providing insights into the complex dynamics underlying MUD severity. The specification of moments, such as mean and variance, was inherent in the bootstrapping procedure, which assumes that the sampling distribution of the indirect effect is adequately approximated by resampling with replacement from the observed data. Through this detailed statistical approach, our study aimed to shed light on the nuanced mechanisms by which early life adversity, through the mediating role of depression and the moderating influence of cognitive function, impacts the severity of methamphetamine use disorder.

## Results

**Table 2** presents the descriptive statistics, neuropsychological results, behavioral measures, and correlations among these variables. This study involved 76 participants, of whom 10.53% were female. The participants had a mean age of 42.17 years and a mean IQ of 89.78. The average ACE score across participants was 4.28. The mean age at initial substance use was 30.51 years, and the average duration of sustained use was 12.59 years. In terms of behavioral measures, the mean Severity of Dependence Scale (SDS), Beck Depression Inventory-II (BDI-II) score, and the average DSM-5 symptom rating by psychiatrists were presented in **Table 2**. For neuropsychological tests, the Iowa Gambling Task (IGT) and the Continuous Performance Test-II (CPT-II) indices were showed. **Table 2** presents the correlations between the variables, with gender correlated with RTSD (Hit reaction time standard Error for CPT-II, r = -.298, *p* <.01), suggesting less consistency in correct reaction time among female participants. Age was correlated with ACEs (r = -.320, *p* <.01), first use age (r = .508, *p* <.01), and sustain use years (r = .306, *p* <.01), suggesting that older cases were of advanced age at the time of their initial substance use than those with younger first use years and less severe experiences of childhood adversity. The IQ was correlated with commission (r = -.350, *p* <.01) and detectability (r = -.307, *p* <.01). Methamphetamine addicts with higher levels of intelligence exhibit an enhanced capacity to discriminate stimuli in the CPT-II.

**Table 2:** Descriptive, behavioral, neuropsychological, and correlation between variables.

Variables	M	SD	1	2	3	4	5	6	7	8	9
1. Gender (F/M)	8/68										
2. Age	42.17	10.13	.121								
3. IQ	89.78	14.71	.024	-.053							
4. ACE	4.28	2.37	-.197	-.320**	.065						
5. First use age	30.51	12.86	.061	.508**	.086	-.353**					
6. Sustain use years	12.59	11.54	.036	.306**	-.139	.117	-.663**				
7. SDS	4.83	3.58	-.089	.033	-.015	.166	-.211	.272*			
8. BDI-II	9.70	10.51	-.022	-.072	-.027	.533**	-.299**	.268*	.231*		
9. DSM-5 symptoms	4.24	1.94	-.158	.000	.062	.398**	-.189	.214	.173	.461**	
10. IGT-total money	-1311.13	831.64	-.091	-.070	-.087	.181	-.046	-.001	-.044	0.24	.178
11. IGT-T score	43.97	6.62	-.081	.172	-.158	-.010	.088	.062	-.077	-.214	.072
12. Omission	61.94	39.47	-.051	.014	-.147	-.041	-.041	.057	-.015	.013	.020
13. Commission	50.02	10.18	.057	-.120	-.350**	.038	-.037	-.069	-.026	.163	-.068
14. Reaction time	53.79	11.99	-.205	.202	-.020	-.098	.167	-.003	-.080	-.149	.154
15. RTSD	49.62	14.68	-.298**	-.085	-.164	.090	-.047	-.018	.045	.011	.214
16. Variability	50.65	14.11	-.187	-.139	-.197	.035	-.137	.035	.145	.042	.215
17. Detectability	50.22	10.42	.112	-.047	-.307**	.032	.029	-.078	.018	.127	-.138
18. Perseveration	57.30	24.60	.127	.029	-.077	.275*	-.157	.203	.073	.318**	.234*
19. Hit RT Block Change	51.89	10.53	-.279	-.075	.073	.028	-.101	.043	.001	.150	.068

*p <.05; **p <.01; IQ, intelligence score; ACE, adverse childhood experiences; First-use age, age when first drug use took place; Sustain use years, total years of using drugs; IGT-total money, mean total money earned in Iowa Gambling task; IGT-T score, T-score in Iowa Gambling task; Omission, t-score of omission index in CPT-II; Commission, t-score of commission index in CPT-II; Reaction time, t-score of reaction time in CPT-II; RTSD, Hit reaction time standard Error of CPT-II; Variability, t-score of variability index in CPT-II; Detectability, t-score of detectability index in CPT-II; Perseveration, t-score of perseveration index in CPT-II; Hit RT Block Change, t-score of hit reaction time of block change in CPT-II; SDS, severity of dependence scale; BDI-II, Beck depression inventory-II.

**Table 2** also indicates that the first use age was correlated with sustain use years (r = -.663, *p* <.01) and BDI-II (r = -.299, *p* <.01). Sustain use years were correlated with SDS (r = .272, *p* <.05) and BDI-II (r = .268, *p* <.05). Concentrating on variables encompassing childhood adversity, cognitive function, depression, and addiction severity levels, ACEs was correlated with first-use age (r = -.353, *p* <.01), BDI-II scores (r = .533, *p* <.01), DSM-5 symptoms (r = .398, *p* <.01), and perseveration of CPT-II (r = .275, *p* <.05). The SDS correlated with the BDI-II (r = .231, *p* <.05). The BDI-II correlated with the symptoms of the DSM-5 (r = .461, *p* <.01) and perseveration of the CPT-II (r = .318, *p* <.01). The symptoms of the DSM-5 were related to perseveration (r = .234, *p* <.05), which not only underscores the association between addiction and the attenuation of individual decision-making (36), depression (64), and inhibitory control capabilities (37) but also substantiates the connection between reduced inhibitory ability and the severity of depression (65). However, concerning cognitive function, this study only examined the correlation between perseveration and childhood adversity.

**Table 1** presents the regrouping by ACEs cutoff (3/4). There were no significant differences in the demographic variables, including sex, age, or intelligence scores, between the SE and ME ACE groups. Similar to the behavioral scale, the subjective severity of MUD behaviors remained insignificant in the subgroup analysis. However, the SE ACE group showed higher depression scores on the BDI-II and more symptoms, as noted by psychiatrists. Regarding cognitive function in the CPT-II, there were differences between the groups in the perseveration of CPT-II. However, no significant differences were found in the remaining factors, including omission, commission, reaction time (RT), and Hit RT Std. Error (RTSD), variability, detectability, and hit RT block changes. There were also no significant differences observed between the two groups in terms of total money and T-scores on the IGT-2, indicating a significant relationship between inhibitory control performance in methamphetamine addicts and their experiences of childhood adversity, while no such relationship was found regarding their decision-making abilities. Based on previous findings, this study employed perseverance as a factor to construct its association with childhood adversity, depression, and addiction severity.

**Figure 1:**
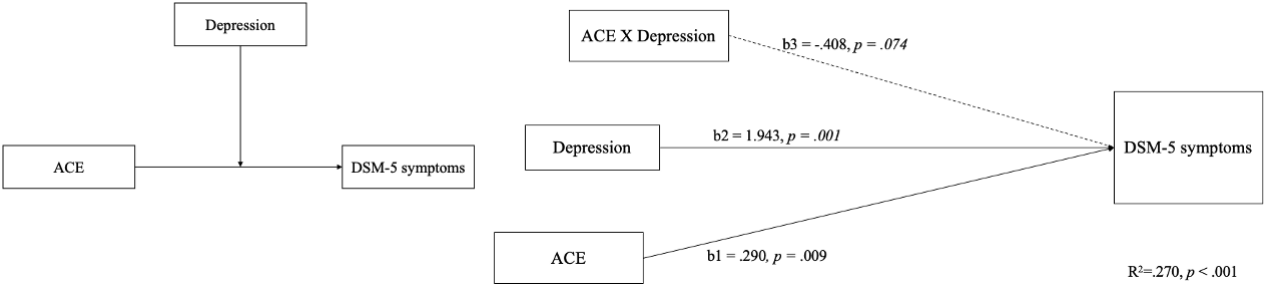
The theoretical and PROCESS model of positing the perseveration capacity of CPT-II only moderates the relationship between depression-modulated ACE scores and SDS scores.

Concerning the symptoms of methamphetamine use diagnosed by psychiatrists, the proposed theoretical framework provides insights into the potential mechanisms by which childhood adversity may precipitate methamphetamine addiction. The relationship between the ACEs, BDI-II, and DSM-5 is central to our overarching research concept. The model templates for the PROCESS by Hayes in Process 4.1, the predictive validity of the model for DSM-5 symptoms, as diagnosed by psychiatrists, was only confirmed for ACEs and BDI-II scores, which explained the severity of DSM-5 symptoms in participants (see **Figure 1**). In this model, significant paths are denoted by solid lines, whereas the dashed line represents a non-significant path. All continuous variables were centered. The R^2^ of model was.270 (p *<*.001), showed a significant path from the independent variable (IV), ACE to the dependent variable (DV), DSM-5 symptoms was shown, b = .290, p = .009; the moderator variable, depression was showed, b=1.943, p *= .*001. however, the interaction between ACE and depression were not shown, b = -.408, p = .074. (see **Figure 1**).

**Figure 2:**
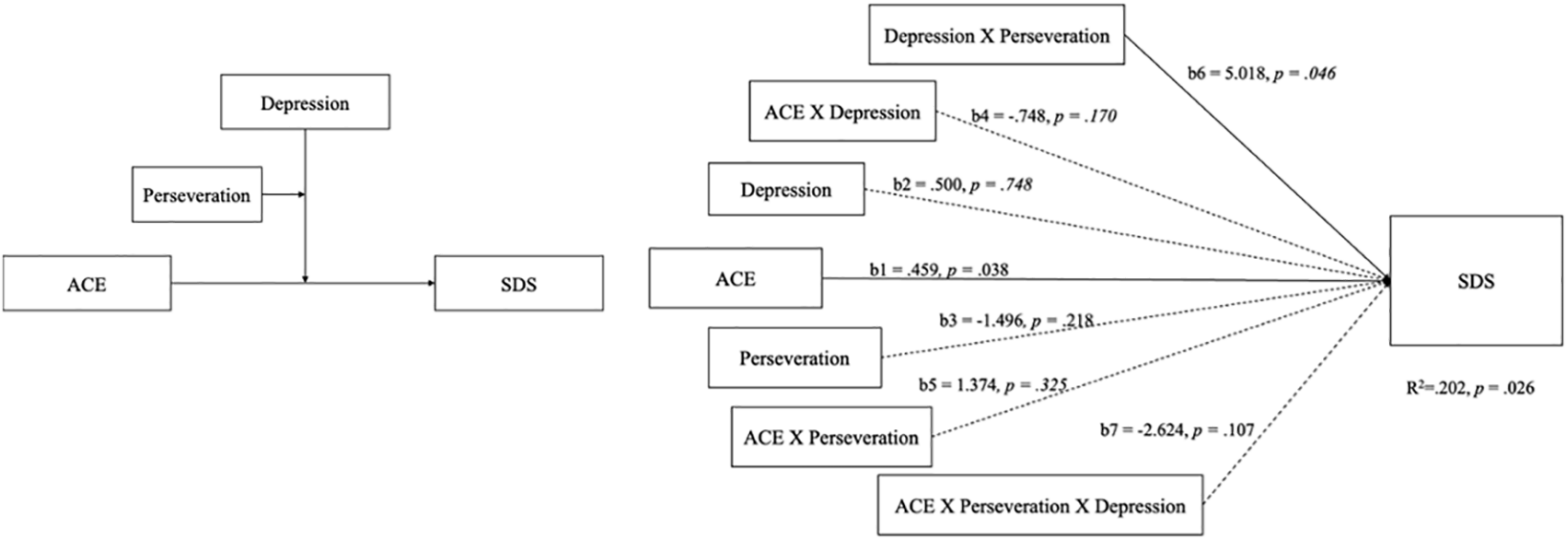
The theoretical and PROCESS model of DSM-5 symptoms scored by psychiatrists was only confirmed for ACE and BDI-II scores, which explained the severity of DSM-5 symptoms in participants.

When considering the interconnections among ACEs, BDI-II, perseveration, and subjective severity symptoms of methamphetamine use, a theoretical model was proposed to elucidate the complex interplay between childhood adversity and ACE-associated factors (see **Figure 2**). Concerning the subjective severity of the symptoms of methamphetamine use symptoms (SDS), the proposed theoretical framework provides insights into the potential mechanisms by which childhood adversity may precipitate methamphetamine addiction. The relationship between the ACEs, BDI-II, and SDS is central to our overarching research concept, as indicated by the aggregate scores on the ACE scale and SDS. The model supports the moderating effect of the interaction between the moderator variables (W and M) on the relationship between the independent variable X and the dependent variable Y. The model accounted for 20.2% of the total variance (p = .026), a significant path from the independent variable (ACE) to the dependent variable (SDS) was shown, b = .459, p = .038. Furthermore, there was significant interaction between the moderators (depression and perseveration), b = 5.0185, p = .046. Thus, we found evidence of moderating effect between depression and perseveration. Which posits that the perseveration capacity of the CPT-II only moderates the relationship between depression-modulated ACE and SDS scores (see **Figure 2**). The model supporting our hypothesis that ACEs influence addictive behaviors through alterations in cognitive function. Based on the above, the interplay between perseveration ability and depression status mediates the subjective severity of SDS. However, this study does not support the notion that ACEs affects non-subjective addiction severity through cognitive processes.

## Discussion

To the best of our knowledge, no study has examined the relationship between the characteristics of methamphetamine addicts and their childhood adversity experiences and has delved into the potential involvement of cognitive function in affecting the association between experiences of childhood adversity and MUD. Compared to the mild exposure (ME) group, the addicts of methamphetamine in the severe exposure (SE) group had a more serious diagnosis by the DSM-5, started using METH at a younger age, had a higher ratio of previous suicide attempts, and higher scores on the BDI-II. The SE group demonstrated more pronounced deficits in inhibitory control, their decision-making abilities remained unaffected. Similar to previous studies demonstrating that METH users exhibit higher ACE scores in comparison to healthy controls in Taiwan (66), this study takes an additional stride by indicating a positive connection between higher ACE scores and the severity of addiction. Other results, such as the ACE scores showed a significantly increased risk of suicidal ideation (67), a higher prevalence of depression and ACEs among methamphetamine users (68), and a positive correlation between ACEs and the duration of METH use (16), results are consistent with those of previous studies. Furthermore, adult substance use such as alcohol consumption is associated with ACEs and worse executive functions, including suppression, emotional control, and self-monitoring (69). Our data provide two novel findings regarding the effect of ACEs on MUD.

First, the use of methamphetamine has been linked to the deterioration of inhibitory control (70–72) and emotional states (73–75), which exacerbate ACEs. Instances categorized as more severe according to the DSM-5 diagnostic criteria were positively associated with increased ACEs. Traumatic events, such as harsh parenting, were negatively associated with inhibitory control in the regression analyses after controlling for the child’s age. Parents who engage in harsh interactions with their children may create an environment with fewer opportunities for the practice and cultivation of executive functions, including inhibitory control (76). The deterioration of inhibitory control directly predicted by ACEs (77, 78) implies a potential reduction in infants’ capacity to utilize this control mechanism to regulate their emotions (79). The attentional control mechanism was preliminarily designed to improve the behavior of infants or children. Therefore, improving attentional control mechanisms could offer either an emotional boost or an adaptable behavior, possibly influencing individuals’ social behaviors or fulfilling their intrinsic needs (80).. This observation could explain why individuals with a higher degree of childhood adversity exhibited a more pronounced severity of methamphetamine use.

Our data confirm that the prediction of subjective severity based on ACEs is subject to moderation by an individual’s inclination toward depressive tendencies. The moderating role of depression in this model differs from that identified by HE et al. (24). Furthermore, it has been indicated that the moderating impact of a propensity toward depression in this model interacts with the individual’s inhibitory control ability. This finding supports the hypothesis that inhibitory control does not directly affect the positive relationship between ACEs and methamphetamine use. Instead, it induces a modification in addictive behavior by influencing an individual’s emotional state. However, the integrative model proposed by Zelazo and Cunningham (81) posits that executive functions, including inhibitory control, interact with emotional regulation, particularly when individuals encounter situations requiring goal-directed problem-solving. When emotion modulation is secondary and resolves other issues, unregulated emotions can impair executive functioning by adding extra strain to an already burdened information processing system, which may directly impair problem-solving abilities (82). From the perspective of executive function, inhibitory control is regarded as the central mechanism of emotional regulation (83), and its development is accelerated during the preschool period (84). Childhood adversity can have a detrimental effect on the development of both inhibitory control and emotion regulation. Future research should clarify the role of emotional states (moderating or mediating effects) in the impact of childhood adversity on addictive behavior.

Second, this study did not provide evidence that childhood adversity influenced the decision-making ability of methamphetamine addicts. Experiences in childhood and adolescence elicit decision-making strategies that are adaptive to prevailing environmental circumstances and can persist into adulthood (85). Early experiences of adversity influence decision-making strategies, which may help individuals adapt to their early caregiving environment (86). In the decision-making process, participants with childhood adversity may experience reduced reward sensitivity and integrate less feedback. Individuals exposed to ACE tend to accumulate fewer rewards from the environment (87). Poor IGT performance could signify increased susceptibility to immediate rewards or diminished sensitivity to probabilistic losses (88), implying that immediate rewards associated with MUD may not significantly impact addictive behavior in individuals with varying childhood adversity experiences. Furthermore, the performance of individuals with SUD on the IGT may not serve as a sensitive indicator of their decision-making capabilities. Not all studies consistently indicate the presence of differences between the initial treatment and the end results (89–91), or between using the substance and not using it (92). Considering the above, this study did not substantiate decision-making ability as a primary factor in comprehending how childhood adversity influences methamphetamine usage patterns.

Although this study provides insights into the potential mechanisms through which childhood adversity influences adult MUD, the current findings are limited by inadequate control of potential confounding variables. First, concerning causality, the relationship between ACEs and subsequent MUD remains unclear as to whether the findings derived from the observational investigations in this study truly signify causal effects. Second, concerning replicability, this study used a cohort of first-time MUD offenders under deferred prosecution in Taiwan. Consequently, the generalizability of these findings to other populations of methamphetamine users or individuals involved in different forms of illicit drug use may be restricted. Third, the severity of the DSM-5 diagnosis was directly predicted by ACEs, but subjective severity was not. The assessment of methamphetamine addiction severity in our model relied on participants’ subjective recollection; this indicator could potentially reflect social alienation or the capacity for emotional and cognitive regulation among addicts (93). Classification of addiction severity according to the DSM-5 criteria by attending psychiatrists did not fit this model. Future studies should examine the influence of various addiction-severity metrics on this pattern. The model can also be refined by including a more direct prediction of biomarkers such as peripheral IL-6 levels (94). Furthermore, cognition-related Electroencephalography may exhibit greater sensitivity to alterations in cognitive function than behavioral manifestations (95). Finally, the explanatory capacity of the theoretical model developed in this study was 20%. This modest explanatory power could be attributed to the complexity of drug addiction, which is influenced by multiple mechanisms and factors (96). Exploring how diverse childhood adversities influence MUD in adults with multiple factors is another subject for future research.

Our hypothesis draws on a broad spectrum of theoretical and empirical foundations suggesting that the effects of ACEs on addiction are mediated through more intricate mechanisms than direct causal pathways. The lack of straightforward correlations between ACEs, cognitive measures, and MUD severity does not negate the potential for ACEs to exert significant indirect effects on addiction through cognitive alterations. Indeed, the complexity of human cognition and behavioral outcomes necessitates a consideration of nuanced relationships that may not be captured through simple correlational analyses. However, we propose that cognitive alterations stemming from ACEs may manifest in specific domains relevant to addiction vulnerability, such as emotional regulation, impulse control, and stress responsiveness, rather than in global cognitive measures that were the focus of our initial analysis. Our analytical approach, incorporating moderated mediation models, allows for the examination of conditional indirect effects that may elucidate the pathways through which ACEs impact MUD severity via cognitive alterations. This approach recognizes the possibility that the relationship between ACEs and addiction severity is contingent upon the presence of mediating variables such as depression, and moderated by other factors including, but not limited to, the individual’s socio-demographic background, the severity and type of ACEs encountered, and concurrent mental health conditions. The absence of significant differences between groups based on ACE exposure in direct measures of cognitive function does not preclude the presence of subtle cognitive impairments that may influence addiction severity. It is possible that these impairments are context-dependent, emerging more prominently in situations that simulate real-life decision-making and stress responses related to substance use rather than in the structured environment of neuropsychological testing. In light of these considerations, future research should emphasize the importance of adopting a holistic view of the impact of ACEs on addiction, which accounts for the mediating role of cognitive alterations and the potential for these effects to be moderated by a range of individual and environmental factors.

In summary, while direct correlations between ACEs, cognitive measures, and MUD severity were not observed in our study, we support our hypothesis through a theoretical framework that acknowledges the complex, mediated, and moderated relationships between developmental trauma, cognitive function alterations, and addictive behaviors. This perspective aligns with current understanding in the fields of developmental psychology and addiction science, advocating for a nuanced approach to studying the long-term impacts of childhood adversity.

## Data availability statement

The raw data supporting the conclusions of this article will be made available by the authors, without undue reservation.

## Ethics statement

The studies involving humans were approved by IRB of Jianan Psychiatric Center, Taiwan. (IRB No: 23-009). The studies were conducted in accordance with the local legislation and institutional requirements. Written informed consent for participation in this study was provided by the participants’ legal guardians/next of kin.

## Author contributions

C-HK: Data curation, Formal analysis, Funding acquisition, Investigation, Methodology, Project administration, Resources, Software, Validation, Visualization, Writing – original draft, Writing – review & editing. Y-CLu: Methodology, Project administration, Resources, Supervision, Validation, Writing – review & editing. C-HL: Conceptualization, Data curation, Formal analysis, Funding acquisition, Investigation, Methodology, Project administration, Resources, Software, Supervision, Validation, Visualization, Writing – original draft, Writing – review & editing. Y-CLi: Conceptualization, Data curation, Formal analysis, Investigation, Methodology, Project administration, Software, Supervision, Validation, Visualization, Writing – original draft, Writing – review & editing.
